# Calibration Test of PET Scanners in a Multi-Centre Clinical Trial on Breast Cancer Therapy Monitoring Using 18F-FLT

**DOI:** 10.1371/journal.pone.0058152

**Published:** 2013-03-13

**Authors:** Francis Bouchet, Lilli Geworski, Bernd O. Knoop, Ludovic Ferrer, Alina Barriolo-Riedinger, Corinne Millardet, Marjolaine Fourcade, Antoine Martineau, Anne Belly-Poinsignon, Francis Djoumessi, Karine Tendero, Laurent Keros, Frederic Montoya, Christel Mesleard, Anne-Laure Martin, Franck Lacoeuille, Olivier Couturier

**Affiliations:** 1 Department of Nuclear Medicine, Academic Hospital, Angers, France; 2 Departments of Radiation Protection and Medical Physics, Hannover Medical School, Hannover, Germany; 3 Department of Nuclear Medicine, R Gauducheau Cancer Centre, Nantes, France; 4 Department of Nuclear Medicine, GF Leclerc Cancer Centre, Dijon, France; 5 Department of Nuclear Medicine, J Perrin Cancer Centre, Clermont-Ferrand, France; 6 Department of Nuclear Medicine, Academic Hospital, Montpellier, France; 7 Department of Nuclear Medicine, Saint-Louis Academic Hospital, Paris, France; 8 Department of Nuclear Medicine, R Huguenin Cancer Centre, Saint-Cloud, France; 9 Department of Nuclear Medicine, Academic Hospital, Tours, France; 10 Department of Nuclear Medicine, Academic Hospital, Bordeaux, France; 11 Department of Nuclear Medicine, Hospital, Bayonne, France; 12 Department of Nuclear Medicine, Curie Cancer Centre, Paris, France; 13 UNICANCER, Paris, France; 14 LUNAM University, Angers University, UMR INSERM 1066 MINT, Angers, France; The University of Chicago, United States of America

## Abstract

A multi-centre trial using PET requires the analysis of images acquired on different systems We designed a multi-centre trial to estimate the value of 18F-FLT-PET to predict response to neoadjuvant chemotherapy in patients with newly diagnosed breast cancer. A calibration check of each PET-CT and of its peripheral devices was performed to evaluate the reliability of the results.

**Material and Methods:**

11 centres were investigated. Dose calibrators were assessed by repeated measurements of a 68Ge certified source. The differences between the clocks associated with the dose calibrators and inherent to the PET systems were registered. The calibration of PET-CT was assessed with an homogeneous cylindrical phantom by comparing the activities per unit of volume calculated from the dose calibrator measurements with that measured on 15 Regions of Interest (ROIs) drawn on 15 consecutive slices of reconstructed filtered back-projection (FBP) images. Both repeatability of activity concentration based upon the 15 ROIs (ANOVA-test) and its accuracy were evaluated.

**Results:**

There was no significant difference for dose calibrator measurements (median of difference −0.04%; min = −4.65%; max = +5.63%). Mismatches between the clocks were less than 2 min in all sites and thus did not require any correction, regarding the half life of 18F. For all the PET systems, ANOVA revealed no significant difference between the activity concentrations estimated from the 15 ROIs (median of difference −0.69%; min = −9.97%; max = +9.60%).

**Conclusion:**

No major difference between the 11 centres with respect to calibration and cross-calibration was observed. The reliability of our 18F-FLT multi-centre clinical trial was therefore confirmed from the physical point of view. This type of procedure may be useful for any clinical trial involving different PET systems.

## Introduction

Evaluation of a reliable quantitative or semi-quantitative index having predictive value is an important issue in clinical PET studies namely for monitoring cancer therapy. In this research area, a national clinical trial was recently promoted by UNICANCER to estimate the value of ^18^FLT-PET for predicting response to neoadjuvant chemotherapy in patients with newly diagnosed breast cancer (ClinicalTrials Identifier: NCT00534274).

In such multi-centre trial, nuclear medicine devices (from dose calibrators to PET systems) come from different manufacturers, have different technical specifications and are used differently according to local practices. These differences may affect PET results, leading to a heterogeneous panel of PET images of different quality and moreover impairing the computation of parametric values, especially the Standardized Uptake Value (SUV). Although SUV is the most available and thus currently used semi-quantitative index in clinical practice and in clinical trials, its accuracy has been widely discussed [1]. Sources of SUV variability are related to biological factors (body size measurement, blood glucose level) and to technologic factors (Uptake time, reconstruction parameters, …) [2]–[4]. In this last category, the calibration error between scanner and dose calibrator is of major importance as well as the SUV definition [5], [6]. As a consequence, SUV estimation could deviate up to 50% in some cases [7].

In the procedure of PET scanner quality control, the calibration is the step establishing the relationship between event rate detected in each pixel and the true activity concentration of the corresponding volume element in the phantom. Usually, calibration is achieved using a phantom provided by the PET system manufacturer according to their own protocol, and has to be repeated regularly to assess performance constancy. The phantom of well-known volume is filled homogeneously with a known activity. This activity is determined by measurement in the local dose calibrator. This type of calibration procedure is highly dependent on local dose calibrator accuracy as well as on manufacturer recommendations (phantom volume, activity to be used, acquisition time, and the accuracy of the corrections to be applied, e.g. attenuation, scatter, randoms, count loss, normalization). Thus, the equivalence of such calibrations from different systems and sites has to be verified.

The first objective of our study was to test a procedure assessing the calibration of PET systems, including all devices of the acquisition chain, which is easily applicable to scanners independent on manufacturer, system and site, thus allowing a direct comparison of different systems. The second objective was to apply this procedure in all 11 sites enrolled in our multi-centre trial. The final objective was to ensure that all PET systems were calibrated to a common standard within acceptable limits.

## Materials and Methods

Two physicists were in charge of all examinations: the local physicist of each of the eleven nuclear medicine departments involved in this study, and the physicist of this national multi-centre clinical trial, who participated in all tests.

### Data acquisition

#### Dose calibrator

One dose calibrator from each site was assessed giving 11 datasets in total. This step was performed first using a solid certified standard source (QSA Global France, Courtaboeuf) of ^68^Ge (50 MBq at the beginning of the study (01/10/2007)). The evaluation consisted in a reproducibility test (placing the standard source 10 times consecutively in the dose calibrator) and an accuracy test.

#### Clock accuracy

the difference between clock associated with the dose calibrator and clock inherent to the scanner was evaluated.

#### PET-CT systems

11 PET-CT scanners (i.e. one per centre) were investigated: two Discovery LS and three Discovery ST (General Electric HealthCare), one Biograph (Siemens) and five Gemini (Philips), one of which used Time-of-Flight technology. Both Discovery LS systems allowed acquisitions in 2D mode only, while from the three Discovery ST two were currently used in both 2D and 3D mode, and one was used in 3D mode only. The Biograph and the five Gemini systems allowed acquisitions in 3D mode only. This resulted in 13 acquisitions: four in 2D mode and nine in 3D mode. All the acquisitions were performed with the same phantom, i.e. a cylinder of 20 cm in diameter and 20 cm length homogeneously filled with activity. The active volume was 5550 mL. The phantom was filled with an ^18^F-FDG activity depending on the acquisition mode used: 300 MBq using 2D mode or 100 MBq using 3D mode, respectively. When the PET scanner was used in both modes, the phantom was prepared for a 2D acquisition, and then the 3D acquisition was performed 2 hours after the end of the 2D acquisition in order to reach activity level required for 3D. Data were acquired during one hour to keep the statistical noise as low as possible. In order to assess the calibration of the scanner on a comparable base, as far as possible it was recommended to reconstruct images with the filtered back-projection algorithm (FBP) utilizing a ramp filter (no apodisation) with Nyquist frequency cutoff, zoom 2, 128×128 matrix, all corrections applied in clinical routine (detector normalization, count loss, CT-based attenuation, randoms and scatter). When filtered backprojection algorithm was not available in clinical mode (Gemini systems), the clinically routine algorithm was used.

### Analysis criteria

Data from all sites were analysed by the same person (i.e. the physicist of the national trial) according to a standardised procedure, which consisted in checking each step of the whole acquisition process.

#### Dose calibrator

the first step of the analysis consisted in assessing the reproducibility of the measurements performed on each dose calibrator. Thus, a repeated-measures ANOVA (PRISM 4.0b, 2004, GraphPad Software, USA) was performed on each set of the 10 measurements performed on each calibrator of each site. Assuming good reproducibility of the measurements made on each dose calibrator, no significant difference was expected. If this hypothesis was confirmed, the mean value of each set of data from each calibrator (called A_calibrator_) was calculated. Then, the accuracy of each dose calibrator was evaluated by calculating the relative difference RD_calibrator_(%) between A_calibrator_ and the calibrated ^68^Ge-source activity A_Ge68_:
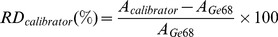
(eq.1)with



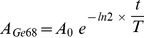
(eq.2)where t is the time difference between the ^68^Ge source calibration date and the date of the dose calibrator test, and T is the half-life of ^68^Ge (270.95 days).

As reported by Geworski et al. [7], a modulus of RD_calibrator_ less than or equal to 10% was considered as the accuracy normally acceptable for this class of instrument.

If the repeated-measures ANOVA resulted in a significant difference between the 10 measurements of the same calibrator, or if the accuracy (RD_calibrator_) was more than 10%, the dose calibrator had to be checked and recalibrated.

In the second step of the analysis the difference between the mean values A_calibrator_ of the 11 dose calibrators and ^68^Ge activities were assessed by a Wilcoxon matched paired test (PRISM 4.0b, 2004, GraphPad Software, USA). As the dose calibrators were measured at different dates and, hence, different ^68^Ge source activities, the Wilcoxon matched paired test was performed on mean values A_calibrator_ normalized by the corresponding ^68^Ge source activity A_Ge68_.

#### Clock accuracy

A difference of less than 2 minutes between calibrator and scanner clocks was considered to be acceptable as it induces an error of less than 1% in activity determination for the half-life of ^18^F. In case of larger differences, the syringe activity measurement had to be corrected for decay.

#### PET system

The measures were performed on the reconstructed images. In order to be independent of the manufacturers, the same software (MIPAV v4.1.2, CIT/NIH, Maryland, USA) was used: 15 cm diameter ROIs were drawn on 15 consecutive slices in the middle of the homogeneous phantom, which represented a total thickness of a minimum of 30 mm on the systems having the smallest slice thickness. The difference between these 15 average activity concentrations was analysed using repeated-measures ANOVA. Assuming good homogeneity in activity concentration in the phantom, no significant difference between the 15 consecutive activity values was expected. If this hypothesis was confirmed, the mean activity concentration for each PET system (called A_PET_) was calculated. Then, the accuracy of each PET system was evaluated by calculating the relative difference RD_PET_(%) between the mean activity concentration of each PET system (A_PET_) and the phantom activity concentration (A_phantom_):
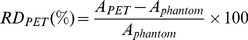
(eq.3)


Furthermore, a relative difference RD_PET,i_(%) was calculated for each slice I of the 15 consecutive slices, and the maximum deviation among the 15 slices was reported for each PET system.

As previously reported [7]–[8], a modulus of RD_PET_ less than or equal to 10% was generally accepted. By extension, a modulus of RD_PET,I_ less or equal to 10% was also accepted. If the repeated-mesures ANOVA resulted in a significant difference between the 15 consecutive activity concentration values of a PET system, and/or a RD_PET_ more than 10%, a new calibration of the PET system was required.

Statistical analysis consisted in assessing the difference between the mean values A_PET_ of the different systems and the corresponding phantom activities with a Wilcoxon matched paired test (PRISM 4.0b, 2004, GraphPad Software, USA). The test was performed for the 2D mode data, for the 3D mode data and for the whole set of data.

A visual analysis of all images was performed, looking for potential artefacts.

For all statistical tests, the significant level was set at 0.05.

## Results

### Dose calibrator

11 dose calibrators were tested (Table 1). For each of them, the repeated-measures ANOVA resulted in no significant difference between the 10 repeated measurements of the standard ^68^Ge source. This result allowed the mean activity A_calibrator_ to be used to evaluate the accuracy by calculating the relative difference RD_calibrator_. As shown in Table 1, all the moduli of RD_calibrator_ were less than 10% (10 out of 11 were lower than 5% and the latest was close to this limit at 5.63%). The Wilcoxon matched paired test resulted in no significant difference between the A_calibrator_ values of the 11 dose calibrators and ^68^Ge activities.

**Table 1:** Dose calibrator reproducibility and accuracy results.

	Measurement #													
Site #	1	2	3	4	5	6	7	8	9	10	Mean	SD	Standard source activity	Accuracy
1	46.15	46.20	46.18	46.13	46.16	46.16	46.16	46.14	46.11	46.12	46.15	0.06	45.48	1,47
2	43.30	43.30	43.30	43.30	43.30	42.90	43.30	43.30	43.30	43.30	43.26	0.29	43.66	−0,91
3	43.70	43.70	43.70	43.70	43.70	43.70	43.70	43.70	43.70	43.70	43.70	0.00	41.37	5,63
4	38.20	37.90	37.80	37.80	37.90	38.00	38.00	37.90	37.80	37.80	37.91	0.34	37.92	−0,04
5	36.98	36.89	36.86	36.89	36.82	36.87	36.82	36.86	36.88	36.81	36.87	0.13	37.25	−1,03
6	34.42	34.34	34.37	34.39	34.42	34.38	34.39	34.37	34.33	34.35	34.38	0.09	32.86	4,61
7	30.77	30.81	30.76	30.77	30.8	30.79	30.75	30.79	30.76	30.76	30.78	0.07	32.28	−4,65
8	12.50	12.50	12.68	12.53	12.52	12.54	12.52	12.51	12.49	12.50	12.53	0.44	12.46	0,59
9	9.90	9.80	9.90	9.90	9.90	9.90	9.80	9.80	9.90	9.90	9.87	0.49	9.87	0,03
10	7.60	7.60	7.60	7.60	7.60	7.60	7.60	7.60	7.60	7.60	7.60	0.00	7.84	−3,02
11	6.90	6.91	6.92	6.94	6.92	6.94	6.91	6.93	6.94	6.91	6.92	0.21	6.97	−0,64

Repeated measurements of certified standard source (^68^Ge) on the dose calibrators of 11 sites. Standard source activity was calculated by applying decay calculation between the calibration date and the measurements date. Measurements expressed in MBq. Standard deviation (SD) and accuracy are in %.

### Clock accuracy

The maximum difference observed between the dose calibrator and PET system clocks in the 11 centres was less than 2 minutes, thus no decay correction was applied for ^18^F.

### Reconstructed images

#### Calibration factors

13 datasets were acquired (four in 2D mode and nine in 3D mode). Because of a missing DICOM tag value on one 3D-dataset (one PET system), the quantitative analysis could not be performed. A new control of this system was not done since this centre has never included a patient.

For each of the 12 other datasets (10 PET systems), the repeated-measures ANOVA resulted in no significant difference between the 15 activity concentration values estimated from 15 consecutive slices (Table 2 and Table 3). This result allowed the mean activity concentration value A_PET_ to be used to evaluate the accuracy by calculating the relative difference RD_PET_. As shown in Table 2, the values of RD_PET_ ranged from −9.97% to +2.30% with a median value of −2.94% in 2D mode, and from −1.67% to +9.60% with a median value of −0.36% in 3D mode: all the moduli of RD_PET_ were less than 10%, and the maximum deviation of RD_PET,I_ among the 15 consecutive slices of each PET system was also less than 10% for all the PET systems. The Wilcoxon matched paired test resulted in no significant difference between the A_PET_ values of the 12 datasets and phantom activity concentrations. Furthermore, as can be seen on results of sites 1 and 4 (Table 2), no relation was observed between 2D deviations and 3D deviations on PET systems working in both modes.

**Table 2:** Mean activity concentration estimated from 15 cm diameter circular Regions of Interest (ROIs) on 15 consecutive slices and accuracy.

	Slice #																	Max deviation	Activity in phantom	Accuracy
Site #	1	2	3	4	5	6	7	8	9	10	11	12	13	14	15	Mean	SD			
1	53.23	52.89	53.08	52.90	53.26	53.07	53.20	53.13	53.44	53.45	55.16	54.81	53.93	53.77	54.17	53.57	1.28	2.97	53.83	−0.49
3	48.20	48.30	48.40	48.44	48.41	48.29	48.13	48.11	48.44	48.61	48.55	48.63	48.60	48.44	48.57	48.41	0.36	−0.62	47.32	2.30
4	46.26	45.95	45.30	45.46	45.39	45.31	45.58	45.85	45.91	46.07	45.98	46.70	46.21	47.12	48.61	46.11	1.86	5.42	51.22	−9.97
7	48.54	48.57	48.53	48.53	48.55	48.59	48.55	48.57	48.63	48.52	48.64	48.77	48.72	48.63	48.48	48.59	0.16	0.37	51.35	−5.38

Data acquired on the 4 PET systems used in 2D mode. Activity concentration in images and in phantom expressed in kBq/mL. Standard deviation (SD), accuracy and maximum deviation are in %.

**Table 3:** Mean activity concentration estimated from 15 cm diameter circular Regions of Interest (ROIs) on 15 consecutive slices and accuracy.

	Slice #																	Max deviation	Activity in phantom	Accuracy
Site #	1	2	3	4	5	6	7	8	9	10	11	12	13	14	15	Mean	SD			
1	27.06	26.38	25.71	25.23	25.21	25.30	25.62	25.88	26.32	26.12	25.30	23.96	23.48	23.81	24.37	25.32	4.06	−7.25	23.10	9.60
2	17.78	17.76	17.73	17.73	17.74	17.71	17.69	17.68	17.69	17.73	17.76	17.74	17.73	17.74	17.74	17.73	0.16	0.30	18.03	−1.67
4	19.12	18.90	18.27	17.81	17.57	17.48	17.58	17.71	17.90	18.25	18.43	18.47	17.51	17.00	16.83	17.92	3.62	6.71	16.97	5.61
5	15.94	15.87	15.85	15.80	15.75	15.78	15.80	15.65	15.70	15.54	15.53	15.61	15.54	15.53	15.41	15.69	0.99	−1.74	15.66	0.17
6	18.04	18.02	18.03	18.03	18.01	17.98	18.00	18.07	18.09	18.04	17.97	17.93	17.93	17.97	18.03	18.01	0.26	0.46	18.17	−0.90
9	17.04	16.75	16.97	16.81	16.82	16.93	16.86	16.83	16.88	16.84	16.84	16.81	16.79	16.63	16.82	16.84	0.56	−1.23	17.07	−1.34
10	18.12	18.12	18.09	18.05	18.05	18.02	18.02	17.96	17.96	17.93	17.89	17.88	17.94	17.96	18.03	18.00	0.43	0.67	17.41	3.40
11	19.74	19.94	20.16	20.48	20.24	20.06	19.92	19.84	19.78	19.70	19.59	19.51	19.41	19.37	19.39	19.81	1.67	3.39	20.10	−1.45

Data acquired on the 8 PET systems used in 3D mode. Activity concentration in images and in phantom expressed in kBq/mL. Standard deviation (SD), accuracy and maximum deviation are in %.

#### Visual inspection

**Figure 1:**
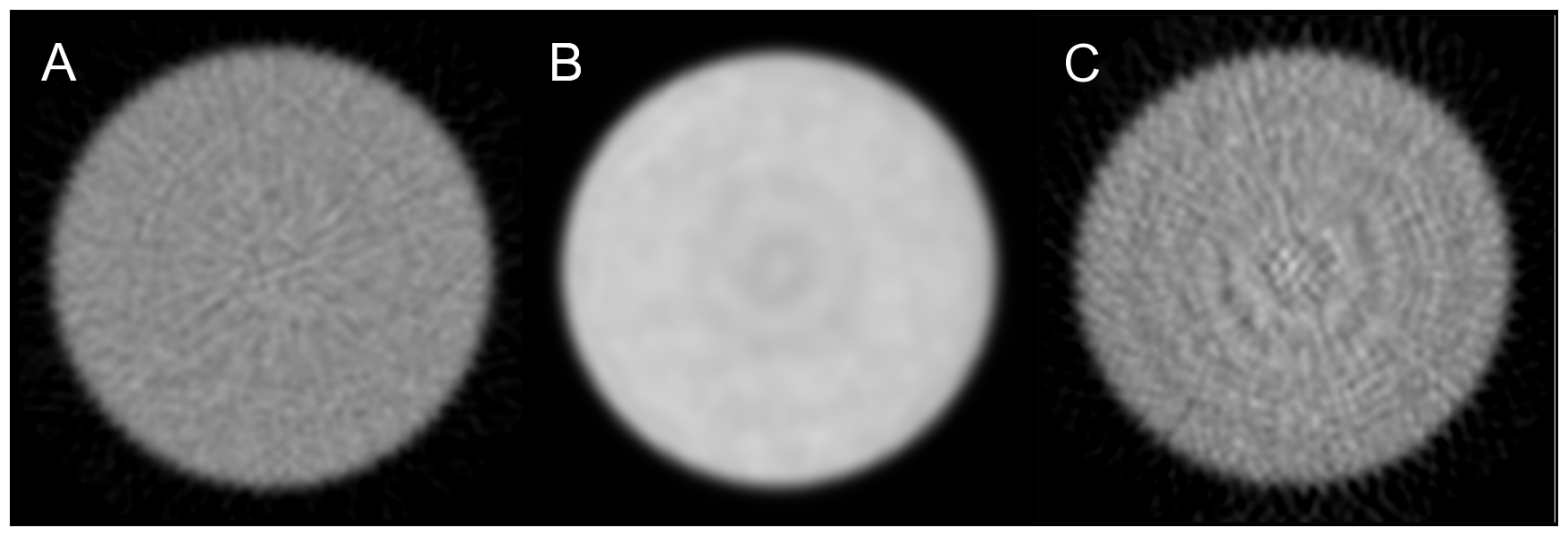
Observed artefacts on images acquired on uniform phantom. Display scale is [0; 100%]. A: no artefacts (site 9), B: LOR-RAMLA concentric ring artefacts (site 6), C: FBP concentric ring artefacts (site 4).

**Figure 2:**
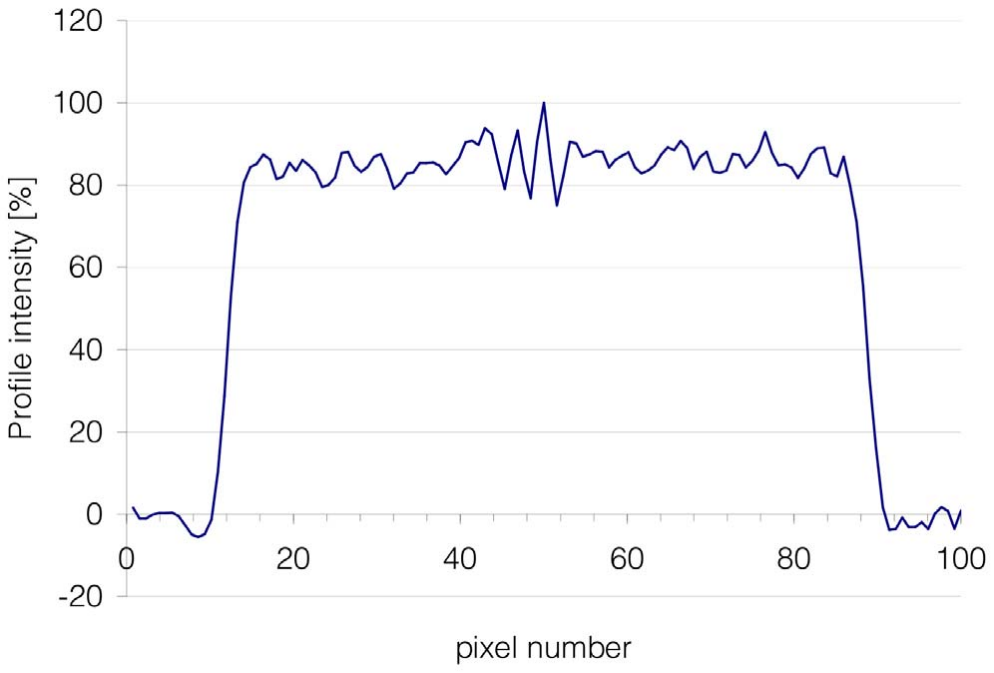
Central profile on a transverse slice of phantoms. Data are normalized to the maximum value intensity of the profile. No abnormality was seen.

Eight datasets showed no artefacts [Figure 1A]. Four datasets showed concentric artefacts in transaxial slices. Horizontal and vertical profiles showed good symmetry for all the 12 datasets [Figure 2].

## Discussion

PET using 2-[^18^F]fluoro-2-deoxy-D-glucose (^18^F-FDG) has become a major player in the field of imaging in oncology and its applications are rapidly expanding, despite ^18^F-FDG having its limitations. Although not likely to replace ^18^F-FDG, emerging data suggest that other fluorinated PET tracers have many potential uses in all phases of the anticancer drug development process, in basic cancer research and in clinical oncology [9]. Over the past two decades, constant efforts have been reported to identify a reliable fluorinated PET tracer able to accurately predict the response to therapy at an early stage, i.e. early during the course of therapy, avoiding side-effects of an ineffective treatment and thus allowing to switch to another. Promising results are expected with 3′-deoxy-3′-fluoro-L-thymidine (^18^F-FLT) [10], [11], namely for monitoring therapy. Indeed, changes in tumour proliferation induced by effective treatment are observed before volume changes since a responding tumour cell will not synthesise new DNA, whereas it may continue to metabolise ^18^F-FDG to maintain different cellular functions.

Clinical studies are required to confirm the potential of a new biomarker, and a large number of patients is most often necessary to obtain reliable qualitative and/or quantitative results. This leads to the design of multicentre clinical trials involving several nuclear medicine departments, permitting rapid patient inclusion which is consistent with the rapid development of research on PET tracers and on PET systems. However, multi-centre clinical trials with a new PET tracer are faced with different problems, the most important being the supply of this new tracer to different and distant nuclear medicine departments. Moreover, in case of protocols designed for the evaluation of tumour response to treatment, the PET tracer needs to be provided at several time-points during the course of therapy, while its production is not always completely reliable, in particular the automated radiosynthesis. We designed a national study to evaluate the potential role of ^18^F-FLT for the determination of the response to anthracycline based neoadjuvant chemotherapy in patients with *de novo* diagnosed breast cancer. In this protocol, several ^18^F-FLT-PET scans were scheduled, i.e. at baseline, after one and after four cycles of chemotherapy, and at the end of treatment before surgery. Missing, for instance, the last ^18^FLT-PET scan due to radiosynthesis failure may invalidate the whole PET dataset acquired in one patient, due to the impossibility to evaluate the response at the end of the neoadjuvant chemotherapy.

Another important issue concerns the consistency of data collected in multi-centre clinical trials, i.e. to ensure the reliability of data acquired in different nuclear medicine departments, on different systems with different data reconstruction methods [10]. For our national study, UNICANCER, the Sponsor of the clinical trial, has approved the development of a quality control procedure to verify that the whole acquisition chain was properly calibrated and to qualify each participating nuclear medicine department. This step is a prerequisite to allow the physicians to quantify PET activity concentration in patients included in our multi-centre clinical trial. To ensure that all PET images were similar in quality as if they had been produced on a single PET system would require much higher standards. This would involve equalizing image contrast recovery (which covers spatial resolution) and matching of signal to noise ratio. However, the tests that were performed in this study covered only the accuracy of the calibration process performed in each participating centre, in order to facilitate analysis of errors, and this is a first prerequisite for pooling of data.

Our approach included all the equipments involved in the final analysis of PET images [12], not only in order to verify the devices themselves, but also to facilitate the identification of errors in the subsequent chain [13]. Thus, our verification process started with a careful dose calibrator checking with a certified ^68^Ge source. Indeed, each PET scanner must be calibrated in terms of activity concentration, allowing the computation of SUV in which a scaling to the injected activity is performed, this activity itself being measured on the dose calibrator. In our study, all the dose calibrators revealed good reproducibility and accuracy [14]. This result is due to the stringent daily controls that have been strengthened by the French regulations and that are performed by radiopharmacists of each nuclear medicine department in charge of the preparation of radiopharmaceuticals.

The dose calibrators and PET system clocks were well synchronised in all centres. In some of them the synchronisation was performed by the local radiophysicist the morning before the checking procedure started. Indeed, the checking procedure guide was sent a few days before and therefore the local radiophysicist sometimes circumvented this assessment. Accordingly, these excellent results of clock synchronisation may not reflect the reality in routine practice, but hopefully operators will now pay attention to the importance of these controls.

Data concerning the PET system calibration were acquired to a high statistical quality to facilitate the detection of systematic errors during subsequent analysis of reconstructed images. The PET system calibration of the 11 centres has shown no major deviation, and the uncertainty of the whole acquisition chain was found to be within 10% tolerance. Nevertheless in some centres, images were generated containing concentric ring artefacts, which had not been identified previously on patient images, probably due to a lower number of events in routine clinical images when compared to the phantom acquisitions. These artefacts appeared on the 3 datasets reconstructed with LOR-RAMLA algorithm but also on one dataset reconstructed with FORE-FBP algorithm. The corresponding CT images were inspected but no circular artefact could be seen on them. A circular artefact in fan geometry should be something that is the same in each re-binned parallel projection for each angle. In our study, as the phantom was centred on the centre of the field of view, the artefacts should be centred on the centre of the re-binned projections. Thus these concentric ring artefacts may be explained by missing or faulty geometric arc correction. Because these concentric ring artefacts were no present on patient images and because no abnormality was seen on profile analysis, concentric ring artefacts were not thought to represent a problem clinically.

Ours results showed that all the ten centres whose images could be analysed could participate in this multi-centre clinical trial without compromising its robustness. The centre whose images could not be analysed because of technical problems did not include any patients in this trial. These results are consistent with the fact that all systems are well monitored with preventive maintenance and regular quality control programs implemented by radiophysicists [15], [16]. PET quality controls are based on manufacturers' and professional associations' recommendations. The regulation should make them mandatory in the near future, but in the meantime it appears necessary to establish specific quality control procedures for all multi-centre clinical trials. These procedures could distinguish two levels: the first level concerning the usual quality controls required by the system manufacturers and mandatory by national regulations, and the second level concerning specific tests for clinical trials, as defined by the investigator. On one hand, as discussed by Geworski et al [7], the daily quality control procedure and visual inspection of images give a rough impression of the PET system's performance, but are not sufficient to validate a system used for quantitative studies. On the other hand, our experience has shown that all the investigated PET systems undergo a more complete monitoring procedure, and that there was an improvement for the subsequent qualification processes of further multi-centre trials, due to training and increasing experience [13].

Our study proposed a simple checking procedure that was quite easily applicable in a few centres, but should be adapted if extended to a large number of centres because of the constraints due to the use of same certified standard source, the same phantom and the necessity of the same operator going to each centre. However, more and more clinical trials are now carried out at a multi-centre level, and require the qualification of each centre. A more complete harmonization is now proposed by the EANM through the EARL program [17], covering contrast recovery equalization and signal to noise ratio matching. Nevertheless, the tests that were performed in the framework of this study appear to be an intermediate step between the simple quality control procedure, which does not guarantee the accuracy of the scanner calibration, and the EARL procedure. In practice, our procedure is a prerequisite for this latter.

## Conclusion

Criteria used to validate the calibration of the PET systems included in our multi-centre clinical trial exhibited an accuracy better than 10% for all sites except one. These first results confirm the fact that multi-centre protocols can lead to results as robust as if acquired on a single system. The whole acquisition chain should be checked regularly either for multi-centre clinical trials or even to assess the constancy of performance in a single centre for the patient's follow-up. Our work showed that a straightforward common procedure for several centres can be established easily. All the sites investigated had a regular quality control program, which could explain the good results observed and could lead assigning the validation procedure to the local physicist before the first patient inclusion.

## References

[1] Boellaard R, Krak NC, Hoekstra OS, Lammertsma A (2004) Effects of noise, image resolution, and ROI definition on the accuracy of standard uptake values: a simulation study. J Nucl Med 45: 1519–1527. 15347719

[2] Paquet N, Albert A, Foidart J, Hustinx R (2004) Within-patient variability of (18)F-FDG: standardized uptake values in normal tissues. J Nucl Med 45: 784–788. 15136627

[3] Adams MC, Turkington TG, Wilson JM, Wong TZ (2010) A systematic review of the factors affecting accuracy of SUV measurements. AJR Am J Roentgenol 195: 310–320 doi:10.2214/AJR.10.4923 20651185 10.2214/AJR.10.4923

[4] Boellaard R (2011) Need for standardization of 18F-FDG PET/CT for treatment response assessments. J Nucl Med 52 Suppl 2 93S–100S doi:10.2967/jnumed.110.085662 22144561 10.2967/jnumed.110.085662

[5] Boellaard R, Oyen WJG, Hoekstra CJ, Hoekstra OS, Visser EP, et al (2008) The Netherlands protocol for standardisation and quantification of FDG whole body PET studies in multi-centre trials. Eur J Nucl Med Mol Imaging 35: 2320–2333 doi:10.1007/s00259-008-0874-2 18704407 10.1007/s00259-008-0874-2

[6] Krak NC, Boellaard R, Hoekstra OS, Twisk JWR, Hoekstra CJ, et al (2005) Effects of ROI definition and reconstruction method on quantitative outcome and applicability in a response monitoring trial. Eur J Nucl Med Mol Imaging 32: 294–301 doi:10.1007/s00259-004-1566-1 15791438 10.1007/s00259-004-1566-1

[7] Geworski L, Knoop BO, de Wit M, Ivancević V, Bares R, et al (2002) Multicenter comparison of calibration and cross calibration of PET scanners. J Nucl Med 43: 635–639. 11994527

[8] Scheuermann JS, Saffer JR, Karp JS, Levering AM, Siegel BA (2009) Qualification of PET scanners for use in multicenter cancer clinical trials: the American College of Radiology Imaging Network experience. J Nucl Med 50: 1187–1193 doi:10.2967/jnumed.108.057455 19525463 10.2967/jnumed.108.057455 PMC2744888

[9] Couturier O, Luxen A, Chatal J-F, Vuillez J-P, Rigo P, et al (2004) Fluorinated tracers for imaging cancer with positron emission tomography. Eur J Nucl Med Mol Imaging 31: 1182–1206 doi:10.1007/s00259-004-1607-9 15241631 10.1007/s00259-004-1607-9

[10] Shields AF, Grierson JR, Dohmen BM, Machulla HJ, Stayanoff JC, et al (1998) Imaging proliferation in vivo with [F-18]FLT and positron emission tomography. Nat Med 4: 1334–1336 doi:10.1038/3337 9809561 10.1038/3337

[11] Been LB, Suurmeijer AJH, Cobben DCP, Jager PL, Hoekstra HJ, et al (2004) [18F]FLT-PET in oncology: current status and opportunities. Eur J Nucl Med Mol Imaging 31: 1659–1672 doi:10.1007/s00259-004-1687-6 15565331 10.1007/s00259-004-1687-6

[12] Geworski L, Knoop BO, Hofmann M, Zander A, de Wit M, et al (2003) [Testing cross-calibration between positron emission tomographs and their peripheral devices]. Z Med Phys 13: 109–114. 12868336 10.1078/0939-3889-00150

[13] Geworski L, Knoop B (2010) Validating PET scanner calibration for multicenter trials. J Nucl Med 51: 997–998 doi:10.2967/jnumed.109.069989 20484431 10.2967/jnumed.109.069989

[14] Geworski L, Karwarth C, Fitz E, Plotkin M, Knoop B (2010) Qualitatskontrolle an PET/CT-Systemen: Erfahrungen und Erfordernisse. Z Med Phys 20: 5–5 doi:10.1016/j.zemedi.2009.10.009 10.1016/j.zemedi.2009.10.009 20304719

[15] Sokole EB, Płachcínska A, Britten A, Georgosopoulou ML, Tindale W, et al (2010) Routine quality control recommendations for nuclear medicine instrumentation. Eur J Nucl Med Mol Imaging 37: 662–671 doi:10.1007/s00259-009-1347-y 20130859 10.1007/s00259-009-1347-y

[16] Boellaard R (2009) Standards for PET Image Acquisition and Quantitative Data Analysis. J Nucl Med 50: 11S–20S doi:10.2967/jnumed.108.057182 19380405 10.2967/jnumed.108.057182

[17] Boellaard R, O'Doherty MJ, Weber WA, Mottaghy FM, Lonsdale MN, et al (2010) FDG PET and PET/CT: EANM procedure guidelines for tumour PET imaging: version 1.0. Eur J Nucl Med Mol Imaging 37: 181–200 doi:10.1007/s00259-009-1297-4 19915839 10.1007/s00259-009-1297-4 PMC2791475

